# Age-Related Diagnostic Accuracy and Patient Acceptance of Two Chewing Efficiency Tests: An Exploratory Field Study

**DOI:** 10.3390/geriatrics11010020

**Published:** 2026-02-16

**Authors:** Alexander Schmidt, Marie-Christin Lehmann, Steffen Schlee, Maximiliane Amelie Schlenz, Bernd Wöstmann

**Affiliations:** 1 Department of Prosthodontics, Christian Albrecht University of Kiel, University Hospital Schleswig-Holstein, Campus Kiel, Arnold-Heller-Strasse 3, 24105 Kiel, Germany; maximiliane.schlenz-helmke@uksh.de; 2 Dental Clinic, Department of Prosthodontics, Justus Liebig University, Schlangenzahl 14, 35392 Giessen, Germany; marie-christin.lehmann@dentist.med.uni-giessen.de (M.-C.L.); bernd.woestmann@dentist.med.uni-giessen.de (B.W.); 3 Department of Geriatric Internal Medicine, Herz-Jesu Hospital Fulda, Buttlarstrasse 74, 36039 Fulda, Germany; s.schlee@herz-jesu-krankenhaus.de

**Keywords:** mastication, dental health surveys, mobile applications, aged, dental prosthesis, sensitivity and specificity, patient acceptance of health care

## Abstract

**Objectives:** This study investigated the impact of age on the diagnostic accuracy and patient acceptance of two chewing efficiency tests: the digital Mini Dental Assessment (MDA) using carrots and the CHEW test by Slavicek using fruit gum, applied in both clinical and nursing home settings. **Methods:** Seventy participants aged 18 to 99 years from dental clinics and nursing homes were included. All participants received a standardized dental examination (reference standard) and performed the MDA and CHEW tests. Sensitivity, specificity, and AUC values were calculated using ROC analysis. Participants rated both tests in terms of taste, consistency, comprehensibility, required time, and subjective chewing sensation. Acceptance was analyzed across age groups and prosthesis types. **Results:** Both chewing efficiency tests showed good agreement with the clinical reference standard. The AUC was 0.72 for the MDA and 0.78 for the CHEW test (*p* = 0.192). Sensitivity was higher for the CHEW test (100%) compared to the MDA (83.3%), while the MDA demonstrated slightly higher specificity (59.6% vs. 55.8%). Age significantly influenced both diagnostic outcomes and test acceptance (*p* < 0.05). Younger participants (<70 years) were more often correctly classified as healthy and tended to prefer the MDA, whereas older participants (≥70 years) preferred the CHEW test, primarily due to taste. Misclassifications occurred most frequently among participants with complete dentures. **Conclusions:** Both the digital MDA and the CHEW chewing test demonstrated good diagnostic performance in identifying treatment need. Acceptance varied significantly with age, suggesting that test selection may be optimized based on patient characteristics. These simple and rapid assessments may support early detection of dental treatment needs in clinical and nursing home settings.

## 1. Introduction

The proportion of older adults in global population is steadily increasing, accompanied by a growing number of individuals who depend on long-term care services in nursing homes [1,2]. Oral health is a key determinant of general health, nutritional status, and overall quality of life in older adults [3,4,5]. However, regular dental visits often decline with increasing age and frailty, leading to delayed diagnosis and treatment of oral diseases [6,7,8]. To address these challenges, practical, rapid, and reliable screening tools are highly needed to support early detection of dental treatment needs—particularly in community and nursing home settings where access to dental care is limited [9,10].

In geriatric care, simple and reliable methods are needed to help caregivers identify individuals who may appear subjectively satisfied with their oral condition but objectively exhibit substantial dental treatment needs [11]. Assessment instruments are well established in nursing for standardized collection of health information, and in dentistry, masticatory function tests have proven particularly useful. These tests provide a practical way to evaluate a patient’s ability to comminute or mix food—an essential indicator of oral function [12,13]. Besides natural test foods, various standardized artificial materials such as silicone cubes, hardened gelatine, or gummy-based test pieces have been developed to ensure reproducible measurement conditions [9,14,15,16,17,18,19,20]. The Mini Dental Assessment (MDA) is a simple, validated tool for evaluating the chewing efficiency and treatment need, traditionally based on chewing a standardized carrot test piece [18,19]. Natural test foods such as carrots are widely used in mastication research because they are inexpensive and easily available, although they may show greater intrinsic variability compared with artificial standardized materials. Recent digital developments have enabled its implementation as a mobile application (App), allowing for use by nursing staff or caregivers with minimal training [9].

An alternative approach is the CHEW test introduced by Slavicek et al., which assesses chewing performance using standardized fruit gums of varying hardness levels [20].

While previous studies have examined the diagnostic accuracy of these methods, little is known about how patient-related factors—particularly age—affect both diagnostic performance and patient acceptance. Moreover, the applicability of digitalized chewing assessments in heterogeneous, mixed-age cohorts, including nursing home residents, has not been systematically evaluated.

Therefore, the aim of this study was to assess the diagnostic performance and patient acceptance of the digital MDA and the CHEW test across different age groups and prosthesis types. The null hypothesis was that neither diagnostic accuracy nor patient acceptance would differ between the two tests or among patient subgroups.

## 2. Materials and Methods

### 2.1. Study Design and Population

The study was conceived as an exploratory field study to evaluate the two chewing efficiency tests under real-world conditions. This approach was chosen deliberately to capture the practical variability of clinical and long-term care environments and to reflect the heterogeneity typically encountered in geriatric oral health assessment. A convenience sample of 70 participants aged 18 to 99 years was included, recruited from four locations in Germany: the Department of Prosthodontics at the Justus Liebig University Giessen, the Marie-Juchacz-Haus and Haus Ulmtal nursing homes (Workers’ Welfare Association Lahn-Dill e.V.), and the geriatric ward of Korbach City Hospital. These recruitment sites were selected to capture a broad spectrum of the patient population, ranging from independent adults receiving care in a dental clinic to older adults residing in long-term care facilities. This sampling strategy ensured inclusion of participants across different age groups and prosthetic situations under authentic clinical and nursing home conditions. Participants were recruited consecutively based on availability and willingness to participate during the study period. No predefined stratification by age groups was applied, as the primary objective of this exploratory field study was to reflect real-world patient distributions rather than to achieve balanced group sizes. The heterogeneous age distribution was therefore considered a methodological strength, allowing assessment of chewing efficiency tests across a broad spectrum of clinical conditions and care settings typically encountered in geriatric dental practice.

All participants provided written informed consent. The study protocol was approved by the local ethics committee (Justus Liebig University Giessen, Ref. No. 291/20) and registered in the German Clinical Trials Register DRKS00024742. All procedures adhered to the principles of the Declaration of Helsinki.

Dental examinations were conducted by one calibrated dentist. Dentures were evaluated according to the California Dental Association (CDA) quality evaluation system, which classifies restorations into four categories based on predefined diagnostic parameters [21]. The clinical dental examination, including CDA-based prosthetic evaluation and assessment of overall dental treatment need, served as the reference standard for evaluating the diagnostic accuracy of the chewing efficiency tests.

Inclusion criteria were an age of ≥18 years and the presence of at least one of the following dental conditions: natural dentition, single crowns or fixed dental prosthesis on natural teeth or implants, removable partial prosthesis, provisional dentures, implant-retained or double crown-retained overdentures, or complete dentures. In cases of mixed restorations, participants were classified according to the least stable (i.e., lowest-functioning) dental prosthesis. Exclusion criteria included drug or alcohol addiction, tube feeding, malignancies, epilepsy, dysphagia, craniomandibular disorders, radiotherapy within the past three years, orthodontic treatment within the past three years, pathological oral symptoms, xerostomia, allergies to test materials, or absence of informed consent. No additional selection criteria regarding age distribution were defined, as age-related differences constituted one of the primary exploratory outcomes of the study.

Due to the exploratory nature of the study, sample size and age distribution were determined by participant availability rather than formal sample size calculation or stratified recruitment.

### 2.2. Chewing Efficiency Tests

#### 2.2.1. Mini Dental Assessment (MDA)

The MDA was performed using a validated digital application that guided the examiner through each assessment step. A standardized carrot piece (cut to approximately 1 × 2 cm) was placed in the participant’s mouth, and the participant was instructed to chew it for 45 s while avoiding swallowing. After completion of the chewing period, the bolus was retrieved, gently separated from excess saliva, and spread onto a flat surface for evaluation.

Chewing performance was assessed by visually comparing the bolus with six calibrated reference images provided within the App, representing increasing levels of fragmentation. The corresponding category was recorded automatically.

The carrot was chosen as test material because it is widely available, inexpensive, and has been used in validated MDA screening protocols [18,19,22]. Standardization was ensured by using a uniform test piece size, a fixed chewing time (45 s), and app-based visual comparison with calibrated reference images. While natural foods may show some variability in texture and moisture, this low-threshold approach was considered appropriate for a pragmatic screening tool intended for real-world clinical and long-term care settings. The same preparation procedure was applied across all recruitment sites to minimize handling-related variability.

In addition to the chewing assessment, participants were asked about the timing of their most recent dental visit and the age of their current dentures or prosthetic restorations, where applicable. These items are integral components of the established MDA scoring algorithm [19] and were automatically incorporated into the total score by the App.

The final MDA score was generated digitally and categorized as follows:

- ≤30 points: rather no dental treatment needed; only routine checkup
- 31–60 points: should be checked by a dentist
- >60 points: need for dental treatment very likely and clinical dental assessment strongly recommended

It should be emphasized that the MDA represents a screening instrument and does not replace comprehensive clinical examination or treatment planning. The scoring categories are intended to identify individuals with a high likelihood of treatment need and to support referral for professional dental evaluation rather than to provide direct therapeutic recommendations. Results were stored anonymously within the App’s internal database. Further methodological details of the MDA protocol can be found in our earlier publication [9].

#### 2.2.2. CHEW Test

The CHEW test (OREHAB MINDS GmbH, Stuttgart, Germany) was conducted following the standardized protocol described by Slavicek et al. [20]. It employs three fruit gums of predefined hardness levels (soft, medium, hard), which allow for a structured assessment of masticatory performance across different material resistances. For each hardness level, participants chewed the fruit gum for 30 s on the right side, left side, and bilaterally, resulting in nine defined chewing sequences per participant. This procedure enables an evaluation of side-specific and bilateral chewing efficiency under controlled and reproducible conditions.

After each chewing sequence, the chewed material was collected and spread evenly onto a standardized evaluation sheet to ensure consistent presentation of particle distribution. The sheets were then processed using a dedicated analysis device (CHEW analysis system, OREHAB MINDS GmbH, Stuttgart, Germany), which scans and digitally assesses the degree of fragmentation. The system automatically uploads the scan to a secure online platform, where quantitative metrics—such as particle count per chewing sequence, chewing side, and hardness level—are generated. Based on predefined normative reference ranges, the software provides a tabulated output accompanied by a descriptive interpretation of chewing performance.

This digital workflow allows for the objective, reproducible quantification of masticatory performance and has been successfully used in dental and geriatric research to standardize assessment of chewing efficiency across heterogeneous populations. The underlying methodology has been described extensively by Slavicek et al. [20] and in a subsequent review [10], which offer detailed background information beyond the scope of this methodological section.

For methodological clarity, the classification of diagnostic outcomes used for the calculation of sensitivity and specificity for each chewing efficiency test is illustrated in Table 1. Healthy and diseased classifications were defined according to the clinical dental examination performed by the investigator and served as the reference standard for all diagnostic accuracy analyses.

### 2.3. Subjective Evaluation of the Chewing Efficiency Tests

Immediately after completing both chewing tests, participants were asked to evaluate the two methods using a structured ranking procedure. Five predefined aspects were assessed: taste, consistency, comprehensibility, perceived required time, and subjective chewing sensation. The selected evaluation criteria were chosen to reflect key determinants of patient acceptance and feasibility of screening procedures, particularly in geriatric and long-term care environments where compliance and tolerability play an important role in successful implementation of diagnostic tools.

For each aspect, participants ranked the two tests by assigning first or second place (ties were not permitted). Responses were recorded in a standardized Microsoft Excel 2016 sheet (Microsoft Corporation, Redmond, WA, USA). To determine overall preference, rankings across all five aspects were aggregated: a test was considered the participant’s preferred method if it received first place in at least three categories. This rule-based aggregation approach was selected to reflect multidimensional user preference rather than reliance on a single evaluation criterion and to improve robustness of preference classification. In addition, preference for individual evaluation criteria, including taste, was recorded as part of the ranking procedure, allowing descriptive analysis of factors contributing to overall test preference.

### 2.4. Statistical Analysis

All statistical analyses were performed using SPSS Statistics (version 29, IBM, Armonk, NY, USA) and Stata (version 16.1, StataCorp, College Station, TX, USA). Descriptive statistics were used to summarize demographic variables, prosthesis types, and test outcomes, including means, standard deviations, and ranges. Normality of distributions was evaluated using Shapiro–Wilk tests and visual inspection of histograms.

Diagnostic accuracy of the MDA and CHEW tests was evaluated by calculating sensitivity, specificity, and area under the receiver operating characteristic curve (AUC), using the clinical dental examination as the reference standard. ROC curves were plotted for both tests, and differences between AUC values were assessed using the χ^2^ test according to DeLong’s method.

Group comparisons were conducted with appropriate methods depending on data distribution and variance homogeneity. Welch’s ANOVA was applied for continuous variables with heteroscedasticity, followed by Games-Howell post hoc tests. When sample sizes were small, Tamhane’s T2 post hoc test was used. Categorical variables, such as prosthesis type, were analyzed using Fisher’s exact test. For age-stratified analyses, participants were grouped into <70 years and ≥70 years. The threshold of 70 years was selected based on commonly applied geriatric age classifications distinguishing younger older adults from more advanced geriatric populations, in whom functional decline in oral health parameters tends to become more pronounced. Previous geriatric research describes individuals aged 65–74 years as relatively functional “young-old”, whereas higher age groups show increasing functional impairment [23].

Subjective evaluation scores were aggregated as described above and compared between age groups using Satterthwaite’s *t*-test for unequal variances. A two-sided significance level of *p* < 0.05 was applied to all tests.

The sample size was based on the number of eligible participants available during the study period (convenience sample), reflecting real-world clinical and nursing home conditions. All analyses followed a predefined statistical analysis plan to minimize data-driven bias.

## 3. Results

### 3.1. Sample Characteristics

A total of 70 participants aged 18 to 99 years (mean 67.6 ± 22.4 years) were included in the analysis. The study population consisted of 40 female (57.1%) and 30 male (42.9%) participants. Younger and middle-aged adults were primarily recruited from the university dental clinic, whereas older participants were mainly recruited from nursing homes and the geriatric ward.

Participants presented with a wide range of prosthetic restorations, including natural dentition, single crowns or fixed dental prostheses on natural teeth or implants, removable partial dentures, provisional dentures, implant-retained or double-crown–retained overdentures, and complete dentures.

### 3.2. Overall Diagnostic Accuracy of the Chewing Efficiency Tests

First, the overall diagnostic accuracy of both chewing efficiency tests was analyzed.

The CHEW test achieved an AUC of 0.78 (95% CI: 0.71–0.85), indicating good diagnostic accuracy, whereas the MDA showed a slightly lower AUC of 0.72 (95% CI: 0.60–0.83). Although the CHEW test demonstrated numerically higher diagnostic accuracy, the difference between the two AUC values was not statistically significant (χ^2^(1) = 1.70; *p* = 0.192).

In terms of test characteristics, the CHEW test demonstrated perfect sensitivity (100%), correctly identifying all participants with treatment need, while the MDA achieved a sensitivity of 83.3%. Conversely, the MDA showed slightly higher specificity (59.6%) compared with the CHEW test (55.8%). These values reflect the overall diagnostic performance visualized in the ROC curves.

The difference between the two AUC values was not statistically significant (χ^2^(1) = 1.7; *p* = 0.192).

**Figure 1:**
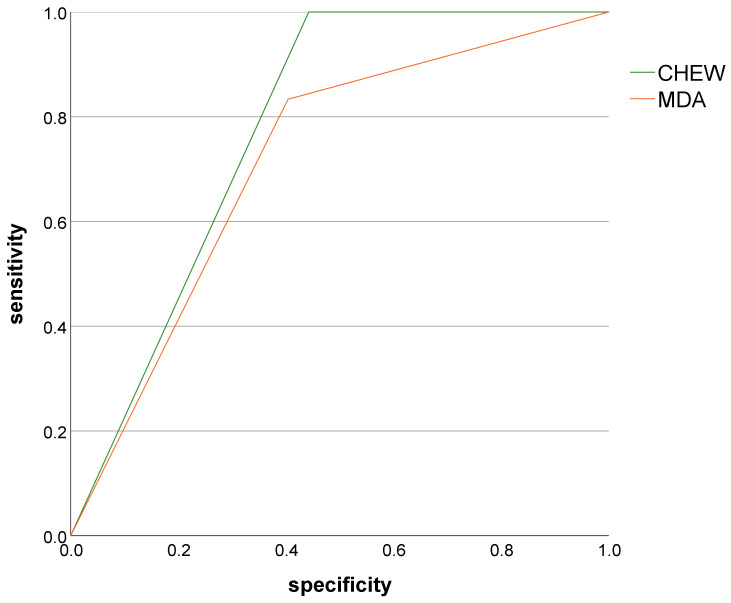
Receiver operating characteristic (ROC) curves for the CHEW test (green) and the Mini Dental Assessment (MDA, orange) using the clinical dental examination as the reference standard.

Sensitivity, specificity, and AUC values for both chewing efficiency tests are summarized in Table 2. Both tests showed good agreement with the clinical reference standard, although their diagnostic strengths differed: The CHEW test demonstrated higher sensitivity (100%), correctly identifying all participants with clinical treatment need. The MDA showed slightly higher specificity (59.6% vs. 55.8%), resulting in fewer false-positive findings among clinically healthy participants. These patterns are reflected in the ROC curves shown in Figure 1 and numerically detailed in Table 2.

Figure 1 displays the receiver operating characteristic (ROC) curves for both chewing efficiency tests in comparison with the clinical reference standard. Both the CHEW test (green curve) and the MDA (orange curve) showed a clear improvement over random classification.

### 3.3. Mini Dental Assessment (MDA) by Age of Participants

The results of the MDA were analyzed as a function of age to evaluate age-related differences in chewing efficiency. For descriptive interpretation, participants were additionally considered in relation to a clinically relevant age threshold of approximately 70 years. Table 3 summarizes the distribution of MDA scores by age and clinical treatment need.

Younger participants (<70 years) consistently exhibited low MDA scores, indicating good chewing efficiency and no need for clinical treatment. Notably, all participants within this age range were clinically healthy and were correctly identified as such by the MDA, with no false-negative findings.

In contrast, older participants (≥70 years) exhibited higher and more variable MDA scores, reflecting reduced chewing efficiency and an increased likelihood of treatment need. Misclassifications—both false-positives and false-negatives—occurred almost exclusively in the older age groups. Participants aged 80 years and older exhibited the highest MDA scores, consistent with the age-related decline in chewing efficiency.

**Figure 2:**
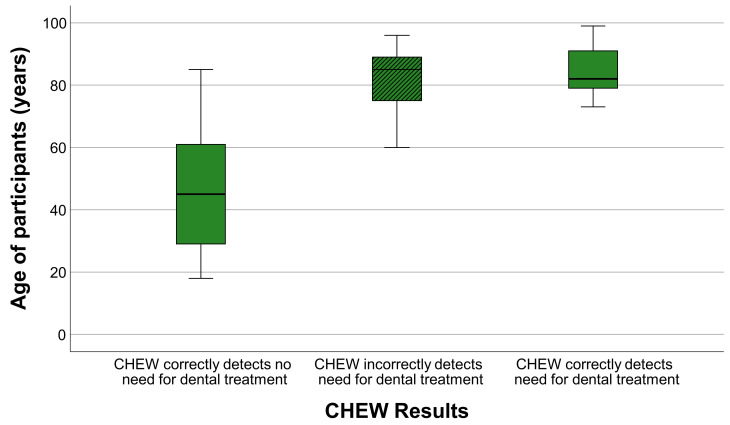
Distribution of MDA scores by age of participants. Incorrect classifications are indicated by hatched boxplots (MDA = Mini Dental Assessment).

These patterns are visualized in the boxplot in Figure 2, which shows the distribution of MDA scores across the ages of participants. The clear increase in scores with advancing age reflects the age-related decline in chewing efficiency captured by the MDA.

While the MDA correctly identified all younger participants (<70 years) as not requiring treatment, it increasingly classified participants in the older age groups as needing treatment. Notably, all individuals under <70 years were clinically healthy and correctly identified by the MDA.

### 3.4. CHEW Test by Age of Participants

The diagnostic performance of the CHEW test was also analyzed across the ages of participants. Table 4 summarizes the distribution of CHEW test outcomes according to age and clinical treatment need.

In younger participants (<70 years), the CHEW test showed high diagnostic accuracy, correctly identifying all clinically healthy participants. As with the MDA, no false-negative results occurred in this age range, indicating that younger participants were reliably classified as not requiring treatment.

In contrast, older participants (≥70 years) exhibited higher CHEW test scores and greater variability, reflecting reduced chewing efficiency and an increased likelihood of classification as needing treatment. Unlike the MDA, the CHEW test produced a notable number of false-positive results in this age group, particularly among individuals with complete dentures. This tendency led to an overestimation of treatment need in older participants, despite the perfect sensitivity of the test.

**Figure 3:**
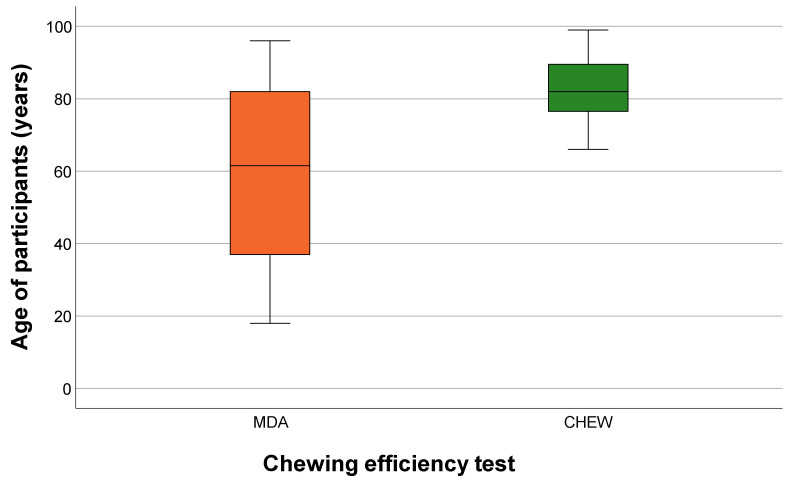
Distribution of CHEW test scores by age of participants. Incorrect classifications are indicated by hatched boxplots (CHEW = CHEW test).

Figure 3 illustrates these age-related patterns: younger participants show tightly clustered, low scores, while older participants display broader score distributions and a shift toward higher values, consistent with age-related decline in chewing ability.

Older participants show greater variability and a tendency toward higher scores, indicating overestimation of treatment need in these groups (CHEW = CHEW test). Overall, while the CHEW test correctly identified all participants with treatment needs (i.e., no false-negatives), it tended to overestimate treatment needs in older adults. A direct comparison of age-related classification patterns between both chewing efficiency tests indicated broadly similar age trends, with increasing misclassification rates in older participants. However, the CHEW test tended to classify older individuals more frequently as requiring treatment, whereas the MDA showed slightly more balanced classification patterns across the age spectrum.

### 3.5. Subjective Evaluation of Chewing Efficiency Tests

Table 5 summarizes the subjective evaluation of the two chewing efficiency tests, based on participant rankings for taste, consistency, comprehensibility, required time, and subjective chewing sensation.

**Figure 4:**
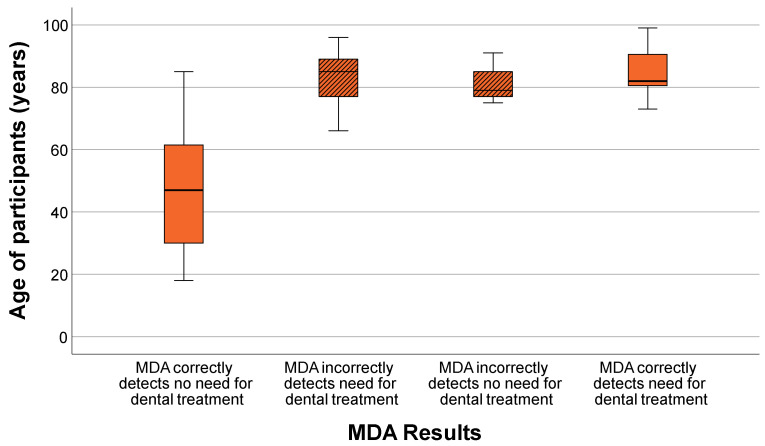
Distribution of preferred chewing efficiency tests by age of participants (MDA = Mini Dental Assessment; CHEW = CHEW test).

The aggregated rankings showed that the majority of participants preferred the MDA overall. However, clear age-related differences were observed. Younger participants consistently favored the carrot-based MDA, whereas older participants increasingly preferred the CHEW test, with taste being one of the most frequently selected favorable evaluation criteria. This age-dependent pattern of preference is further illustrated in Figure 4, which depicts the distribution of preferred tests across ages. A significant age effect was observed (*p* < 0.05), indicating that taste and texture played a major role in subjective acceptance of the tests.

Statistical analysis revealed a significant association between age and the subjective evaluation of the two chewing efficiency tests (*p* < 0.05). While preference for the MDA was evident across all ages, and all younger participants preferred the MDA, participants aged 66 years and older increasingly favored the CHEW test. Accordingly, the null hypothesis had to be partially rejected, as significant differences in acceptability were observed between the two tests.

## 4. Discussion

This exploratory field study examined the diagnostic accuracy and acceptance of two chewing efficiency tests—the Mini Dental Assessment (MDA) and the CHEW test—in an exploratory field study with a heterogeneous population ranging from young adults to very old individuals in clinical and nursing home settings. Both tests showed good agreement with the clinical reference standard, although their performance differed across participant age. In addition, clear age-dependent differences in subjective acceptance were observed, underscoring the importance of selecting assessment tools that align with the functional abilities and sensory preferences of different patient groups.

### 4.1. Diagnostic Accuracy of the Chewing Efficiency Tests

Both chewing efficiency tests demonstrated good diagnostic agreement with the clinical reference standard, although their diagnostic characteristics differed. The CHEW test showed slightly higher diagnostic accuracy compared with the MDA, although the difference was not statistically significant. These results suggest that both methods may provide clinically meaningful information when used as screening tools for estimating dental treatment need [10,24].

The diagnostic performance of the MDA in this study aligns well with previous findings. Wöstmann et al. [19] reported an AUC value of 0.81, and Mausbach et al. [22] found an AUC of 0.83 using the traditional carrot-based MDA test. However, in the latter study, sensitivity was substantially lower (52.3%) and specificity higher (77.1%) compared to the present study. This discrepancy likely reflects differences in the underlying study populations, as Mausbach et al. [22] examined individuals attending routine annual check-ups, representing a more dentally healthy and thus oversampled cohort.

The CHEW test has been less extensively validated in the literature. Previous studies often focused exclusively on younger adults or older adults without providing a comparison across different ages [22]. The CHEW test demonstrated very high sensitivity, indicating a low likelihood of missing individuals with treatment need. This high sensitivity indicates that the test is unlikely to miss individuals who require dental attention, making it suitable for early detection and preventive screening. However, this benefit was accompanied by a higher rate of false-positive classifications, particularly among older adults. Clinically, this may be acceptable in settings where the priority is not to overlook treatment need, even at the cost of recommending additional examinations. Several methodological and material-related factors may contribute to the observed differences in diagnostic performance between the two tests. The CHEW test uses standardized fruit gums with predefined hardness levels and digitally assisted particle analysis, which allows highly controlled and reproducible assessment conditions. In contrast, the MDA relies on a natural test food and visual classification of fragmentation, which may introduce greater variability but reflects a pragmatic screening approach suitable for low-threshold screening in clinical and long-term care settings. These inherent methodological differences may partly explain the higher sensitivity observed for the CHEW test and the slightly higher specificity of the MDA.

Overall, both chewing efficiency tests demonstrated diagnostically useful performance, but their differing sensitivity/specificity profiles suggest that the choice of test may be tailored to the clinical setting: the CHEW test may be advantageous in preventive contexts prioritizing sensitivity, whereas the MDA may be beneficial in populations where reducing false-positives is desirable. Importantly, the MDA is designed as a screening tool rather than a diagnostic instrument for treatment decision-making. High scores indicate an increased probability of dental treatment need or functional impairment and should therefore prompt further clinical evaluation. Final therapeutic decisions require comprehensive dental examination and individualized treatment planning.

### 4.2. Age-Related Differences in Chewing Efficiency

Clear age-dependent differences in chewing efficiency were observed in both chewing efficiency tests. Younger participants showed uniformly low test scores and were consistently classified as not requiring treatment, whereas older participants—particularly those aged 70 years and above—exhibited substantially higher and more variable scores. These patterns are consistent with the well-documented age-related decline in oral function, which is influenced by morphological changes, reduced muscular strength, and increasing dental morbidity. The use of 70 years as an age threshold in the present study is consistent with commonly applied classifications in geriatric research, which distinguish between younger and more advanced older age groups with increasing functional vulnerability [23].

The present findings are consistent with previous research. Slavicek et al. [20] reported markedly better chewing performance in younger adults aged 17 to 51 years and a high degree of interindividual variability even within younger cohorts. Similar trends have been described in studies by Murakami et al. [25] and Igarashi et al. [26], although these investigations focused primarily on older individuals and did not include younger comparison groups. Consequently, the current study provides a broader age-diverse perspective and demonstrates that both the MDA and the CHEW test are sensitive to age-related functional decline.

Physiological explanations support these observations. Van der Bilt [27] demonstrated a strong association between masticatory muscle force and particle comminution efficiency, indicating that reduced muscle function in older adults contributes to higher test scores. In addition, cumulative tooth loss, compromised prosthesis stability, and age-related neuromuscular changes may further reduce chewing capacity in advanced age [24,28].

Testing chewing efficiency in very old individuals also introduces practical challenges. Despite standardized instructions, partial swallowing of test material cannot be fully avoided, and cognitive impairment may affect compliance and self-reported elements of the MDA. Although overt dementia was not intentionally included, undetected cognitive decline in some participants cannot be ruled out. This represents an inherent limitation in geriatric mastication research and may contribute to the heterogeneity of test scores in the oldest age groups.

### 4.3. Subjective Evaluation of Chewing Efficiency Tests

Clear age-related differences were also observed in the subjective evaluation of the two chewing efficiency tests. While the majority of younger participants preferred the carrot-based MDA, older adults showed a marked preference for the CHEW test, with taste frequently ranking among the most favorable evaluation criteria reported by participants. This finding suggests that sensory factors such as taste and texture may play an important role in test acceptability and may outweigh objective chewing efficiency in older individuals [8,29]. Patient acceptance is considered a critical determinant of feasibility and implementation success of screening instruments, especially in frail or institutionalized older populations. Sensory perception, including taste, may influence willingness to complete chewing tests and therefore directly affect the reliability and practical applicability of such screening tools.

Previous studies support these observations. Mausbach et al. [22] reported lower acceptance of the CHEW test among participants with removable dental prosthesis, which may have been influenced by prematurely terminating the test when participants were unable to chew the softest fruit gum. In contrast, the present study administered all hardness levels regardless of initial chewing performance, which may explain why many older adults rated the CHEW test more favorably despite reporting difficulties with the softer variants. Taste preference therefore appears to be a key determinant of subjective acceptance, particularly in older populations.

High acceptance of the carrot-based MDA test across a wide age range has been described previously by Nguyen et al. [30] and Wöstmann et al. [18], who reported that carrots are generally well tolerated and easy to handle in clinical and non-clinical settings. The present findings are consistent with these observations: younger participants uniformly preferred the MDA, whereas older participants increasingly selected the CHEW test for sensory reasons rather than functional ease.

Findings from gerontological nutrition research further support these patterns. Müller and Nitschke [31] reported that with increasing age, individuals tend to prefer softer, stickier, and sweeter foods over hard or fibrous textures. This behavioral shift is reflected in the present study, where older participants showed a clear preference for the sweet CHEW test, independent of their objective chewing efficiency. Such age-related sensory and texture preferences likely contribute substantially to the acceptance of chewing efficiency tests in geriatric populations.

These results highlight an important consideration for clinical implementation: the most diagnostically accurate test may not necessarily be the most acceptable to all participants. Particularly in frail or institutionalized older adults, test acceptance may be shaped by hedonic preferences and prior experiences with food textures. Such factors should be considered when selecting chewing efficiency tests in geriatric settings.

### 4.4. Practical Accessibility and Feasibility

Beyond diagnostic performance and patient acceptance, practical accessibility represents a key factor for the implementation of masticatory function tests in routine geriatric care. The MDA offers a distinct advantage in this regard, as it requires only a standardized carrot test piece and a freely available mobile application, which can be downloaded without cost from common app platforms (e.g., Google Play Store [32] and Apple App Store [33]). This simplicity enables broad application by dental professionals, nursing staff, or caregivers, even in resource-limited settings such as nursing homes or home care environments. In contrast, the CHEW test relies on standardized fruit gums and requires dedicated evaluation infrastructure, including digital analysis via a proprietary system. While this allows for detailed and objective assessment of chewing performance, it may limit widespread implementation due to material costs, device availability, and logistical requirements. Importantly, these differences do not reflect limitations in diagnostic validity but rather illustrate that both methods address different clinical needs: the MDA is particularly suited for low-threshold, rapid screening in everyday care, whereas the CHEW test may be advantageous in settings where more detailed functional analysis is feasible. Consideration of such practical aspects is essential when selecting masticatory function assessments for geriatric populations.

### 4.5. Strengths and Limitations

This study has several notable strengths. It included a broad and clinically relevant age range extending from young adults to frail older individuals in long-term care, thereby reflecting real-world conditions under which chewing efficiency tests are typically applied. The resulting age distribution was deliberately heterogeneous and non-uniform, mirroring the practical realities of recruitment in both clinical and nursing home environments rather than an artificial, age-stratified study design. The use of a digital MDA platform and standardized CHEW test protocols ensured consistent and reproducible testing procedures. Furthermore, combining objective dental findings with subjective participant evaluations offers a multidimensional view of the clinical applicability and acceptability of both methods. Certain methodological considerations should also be noted. As the study was conducted under real-life conditions in both clinical and nursing home settings, participation was inevitably influenced by practical and ethical constraints. Some very old or highly dependent individuals, particularly those with advanced cognitive impairment or medical contraindications, could not be included, which reflects the realities of research in long-term care environments [1,3,4,34,35,36,37]. Minor swallowing of test material during chewing assessments cannot be completely prevented, although instructions and supervision minimize this effect. Finally, the sample size was determined by feasibility within the study period, which is typical for studies conducted in geriatric care settings. The relatively small sample size may have limited statistical power and therefore increases the risk of overlooking potentially relevant group differences. Consequently, the findings should be interpreted as exploratory and hypothesis-generating rather than confirmatory. Given the exploratory nature of the study and the limited sample size, age distributions were primarily described descriptively. Mean age values were presented for overview purposes but should be interpreted cautiously, as age distributions were heterogeneous across classification categories. Larger multicenter studies are required to validate the present findings and to further investigate age-dependent diagnostic performance and acceptance patterns.

Participants were recruited from heterogeneous clinical and care settings, including a university dental clinic, nursing homes, and a geriatric hospital ward. These settings may differ with regard to oral health status, access to dental care, and functional health characteristics, which may have influenced test outcomes. However, this heterogeneity also reflects real-world clinical conditions and was considered appropriate for the exploratory design of the study. Overall, these considerations are characteristic of research involving vulnerable older populations and do not diminish the validity of the main findings.

### 4.6. Clinical Implications and Future Directions

Both the MDA and the CHEW test showed good diagnostic agreement with the clinical reference standard, suggesting their potential use as simple and time-efficient screening tools for identifying dental treatment needs, particularly in settings where comprehensive oral examinations are difficult to implement, such as nursing homes or geriatric wards. Both tests are intended as screening tools supporting identification of individuals who may benefit from further comprehensive dental assessment. The high sensitivity of the CHEW test may help prevent underdiagnosis, whereas the higher specificity of the MDA may reduce unnecessary referrals in younger or predominantly dentate populations.

The present study provides novel insights by combining diagnostic accuracy assessment with patient acceptance across a broad age spectrum. While previous studies have typically evaluated chewing efficiency tests either from a functional or methodological perspective, the current findings highlight the clinical relevance of age-dependent acceptance as an additional determinant when selecting screening tools. These results suggest that screening strategies in geriatric and long-term care settings may benefit from tailoring test selection not only to diagnostic performance but also to patient tolerability and compliance.

Given the known influence of tooth loss and prosthetic rehabilitation on masticatory function, future adaptations of chewing tests may benefit from adjusting scoring thresholds or test materials for individuals with removable dentures, especially complete dentures. In addition, expanding digital tools, such as automated scoring algorithms within the MDA app, may further enhance accessibility, standardization, and usability in clinical and long-term care environments [9,38]. Larger multicenter studies are recommended to validate the present findings and to explore the integration of masticatory assessments into routine geriatric oral health screening programs. While the present findings provide important insights into age-related diagnostic performance and acceptance of chewing efficiency tests, they should be interpreted as exploratory and hypothesis-generating. Larger confirmatory studies are required to validate these findings and to further investigate their applicability in different clinical and care settings.

## 5. Conclusions

In conclusion, this exploratory study suggests that both the digital Mini Dental Assessment and the CHEW test may offer clinically meaningful information on masticatory performance and dental treatment need across a broad age spectrum. Their diagnostic capabilities, combined with age-associated differences in patient acceptance, highlight their value as practical and accessible tools for oral health screening in both general and geriatric care. By providing rapid, user-friendly assessments that can be administered even outside the dental clinic, these methods support early identification of treatment needs and may ultimately contribute to improving oral health outcomes in aging populations. These findings underline the importance of considering patient acceptance alongside diagnostic performance when implementing chewing efficiency screening tools, particularly in geriatric and institutional care populations.

## Figures and Tables

**Table 1:** Structure of the four-field table used for calculating sensitivity and specificity for both chewing efficiency tests. Healthy and diseased classifications were based on the clinical dental examination performed by the investigator and served as the reference standard. True positives (TP) = treatment need correctly identified; False negatives (FN) = treatment need incorrectly classified as healthy; True negatives (TN) = healthy participants correctly identified; False positives (FP) = healthy participants incorrectly classified as requiring treatment.

		Chewing Efficiency Test (MDA/CHEW Test)	
		healthy	diseased
Reference Findings: Dental examination by investigator	healthy	true-negative (TN)	false-positive (FP)
	diseased	false-negative (FN)	true-positive (TP)

**Table 2:** Diagnostic accuracy values for the MDA and CHEW test, including AUC, sensitivity, specificity, and 95% confidence intervals. (MDA = Mini Dental Assessment; CHEW = CHEW test; AUC = area under the ROC curve; SE = standard error; CI = confidence interval. Sensitivity and specificity were calculated using the clinical dental examination as the reference standard. AUC differences were tested using DeLong’s χ^2^ method.

Chewing Efficiency Test	*N*	AUC	SD	95% CI (Lower)	95% CI (Upper)	Chi^2^	*p*-Value
MDA	70	0.72	0.06	0.60	0.83	1.70	0.192
CHEW	70	0.78	0.04	0.71	0.85		

**Table 3:** Distribution of MDA scores by age and clinical treatment need (MDA = Mini Dental Assessment; N = number; SD = standard deviation; Min. = minimum; Max. = maximum).

	Age of Participants (Years)				
MDA Results	N	Mean	SD	Age Range (Min.)	Age Range (Max.)
MDA correctly detects no need for dental treatment	31	47.7	18.3	18	85
MDA incorrectly detects need for dental treatment	21	83.1	8.2	66	96
MDA incorrectly detects no need for dental treatment	3	81.7	8.3	75	91
MDA correctly detects need for dental treatment	15	84.6	7.2	73	99
Total number of participants	70	67.6	22.4	18	99

**Table 4:** Distribution of CHEW test results by age of participants and clinical treatment need. (CHEW = CHEW test; N = number; SD = standard deviation; Min. = minimum; Max. = maximum).

	Age of Participants (Years)				
CHEW Results	N	Mean	SD	Age Range (Min.)	Age Range (Max.)
CHEW correctly detects no need for dental treatment	29	46.5	18.3	18	85
CHEW incorrectly detects need for dental treatment	23	81.4	9.6	60	96
CHEW incorrectly detects no need for dental treatment	0	/	/	/	/
CHEW correctly detects need for dental treatment	18	84.1	7.2	73	99
Total number of participants	70	67.6	2.4	18	99

**Table 5:** Subjective evaluation of chewing efficiency tests by participants (MDA = Mini Dental Assessment; CHEW = CHEW test; N = number; SD = standard deviation; Min = minimum; Max = maximum). Values describe participant age characteristics stratified by overall preferred chewing efficiency test, determined by aggregated ranking across five subjective evaluation criteria. (taste, consistency, comprehensibility, required time, and subjective chewing sensation).

Participant Age Characteristics by Overall Preferred Chewing Efficiency Test					
Chewing Efficiency Test	N	Mean	SD	Age Range (Min.)	Age Range (Max.)
MDA	46	60.0	23.7	18	96
CHEW	24	82.3	8.1	66	99
Total number	70	67.6	22.4	18	99

## Data Availability

The datasets of this article are available from the corresponding author on a reasonable request.

## Abbreviations

The following abbreviations are used in this manuscript:

AUC	Area under curve
CHEW	CHEW chewing efficiency test
CI	Confidence interval
Max	Maximum
MDA	Mini Dental Assessment
Min	Minimum
N	Number
ROC	ROC curve
SD	Standard deviation
SE	Standard error
